# The complete mitochondrial genome of a euphausiid species: *Pseudeuphausia sinica* (Euphausiacea: Euphausiidae)

**DOI:** 10.1080/23802359.2019.1617045

**Published:** 2019-07-10

**Authors:** Xiao Wang, Xuelei Zhang, Fengrong Zheng, Meijie Jiang

**Affiliations:** a Key Laboratory of Science and Engineering for the Marine Ecological Environment, the First Institute of Oceanography, Ministry of Natural Resources, Qingdao, China;; b Laboratory of Marine Ecology and Environmental Science, Pilot National Laboratory for Marine Science and Technology (Qingdao), Qingdao, China

**Keywords:** *Pseudeuphausia sinica*, mitochondrial genome, Class Malacostraca, phylogenetic analysis

## Abstract

We describe the mitogenome sequence of *Pseudeuphausia sinica* collected in the adjacent waters of the Yangtze River Estuary. The assembled mitogenome is 16,192 bp in length and consists 13 protein-coding genes, 22 transfer-RNA genes, 2 ribosomal-RNA genes, and 1 non-coding region. The most common start codon for 13 PCGs is ATG and the most common termination codon is TAA. The overall G + C content was only 28.26% in the heavy strand. The result of phylogenetic analysis showed that the relationship of *P. sinica* was close to the species in the same order.

*Pseudeuphausia sinica* is a common euphausiid species in coastal waters of East China Sea and western Yellow Sea, which was first described by Wang and Chen (1963). The species belongs to the Family Euphausiidae and the Order Euphausiacea. Due to the appropriate bait source of commercial fishes (Yan et al. 2006), and being one of the dominant zooplankton species of Lvsi fishing grounds (Yu et al. 2013), *P. sinica* could be important to the local fishery resource supplement. Previous studies mainly focused on the individual development (Wang 1965; Li et al. 1994), distribution (Chen et al. 2008), and the effects of environmental conditions on its population characteristics (Tao et al. 2013). Less research is relative to the sequence of the mitochondrial DNA genes (Lin et al. 2004).

In the present study, we collected samples of *P. sinica* in northeast of the Yangtze River Estuary (122°30.8′E, 31°30.2′N; 122°59.6′E, 31°30.6′N) in October 2018 with WP2 net (mesh size: 200 μm). The sample was immediately frozen in –80 °C on board until it was picked out under stereo microscope (Leica S8APO) for mitogenome analysis. The mtDNA was sequenced by Illumina Hiseq 4000. Some samples of *P. sinica* collected at the same two stations were preserved in 5% formalin solutions and stored in the plankton laboratory of the First Institute of Oceanography, Ministry of Natural Resources. At present, we describe the complete mitochondrial genome of *P. sinica*, which will help to understand the phylogenetic status of genus *Pseudeuphausia* among the Class Malacostraca and Phylum Arthropoda.

The length of the complete mitochondrial genome of *P. sinica* is 16,192 bp with the GenBank accession No. MK579299. The genome contains 37 genes, including 13 protein-coding genes (PCGs), 22 tranfer RNA (tRNA) genes, 2 ribosomal RNA (rRNA) genes, and 1 control region (D-loop) which is 1518 bp in length. For the 13 PCGs, the most common start codon is ATG (ATP6, COX3, ND4, ND4L and Cytb), then is ATA (COX2, ATP8, ND1 and ND6); the most common termination codon is TAA (COX1, ATP6, ND1, ND2, ND4L and ND6), then is the incomplete termination codon T–– (COX2, COX3, ATP8 and ND4).

The mitochondrial base composition is A 38.37%, T 33.36%, G 11.67%, and C 16.59% in the heavy strand, with an obvious (A + T) % > (G + C) %. Similar situation occurred in the non-coding region (D-loop) in which the (A + T) % was more than 80%.

The phylogenetic relationship were estimated using the Maximum Likelihood method in RAxML 8.1.5. It is showed that the phylogenetic relationship of *P. sinica* is very close to the two species in the Family Euphausiidae: *Euphausia pacifica* and *E. superba*. Meanwhile, the phylogenetic relationship of *P. sinica* is far away from *Cypridopsis vidua* and *Calanus hyperboreus*, which are not the species of Class Malacostraca (Figure 1).

**Figure 1.**
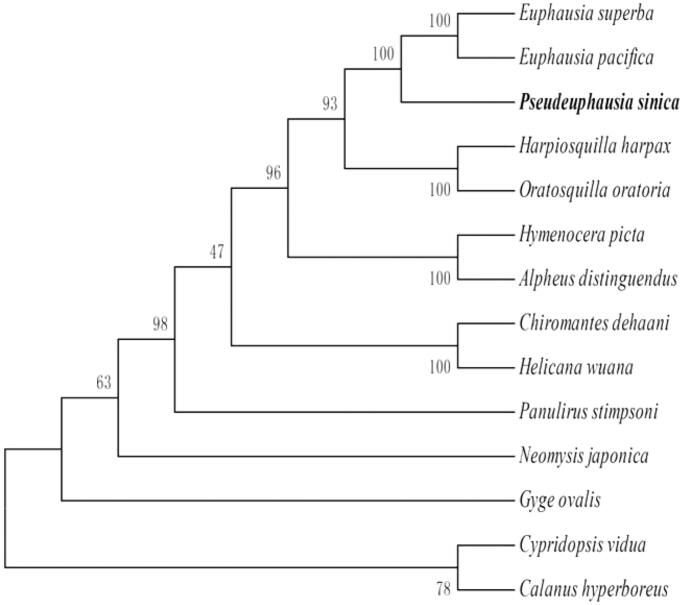
Phylogenetic relationship of 13 species in Phylum Arthropoda based on the concatenated data set of 13 protein-coding genes. Genbank accession Numbers: *E. superba* (NC016184.1), *E. pacifica* (EU587005.1), *Hymenocera picta* (NC039631.1), *Oratosquilla oratoria* (GQ292769.1), *Hymenocera picta* (NC039631.1), *Alpheus distinguendus* (GQ892049.1), *Chiromantes dehaani* (MH593563.1), *Helicana wuana* (MH593562.1), *Panulirus stimpsoni* (GQ292768.1), *Neomysis japonica* (NC_027510.1), *Gyge ovalis* (NC037467.1), *C. vidua* (NC028407), and *C. hyperboreus* (NC019627.1).
